# Achieving Conservation Science that Bridges the Knowledge–Action Boundary

**DOI:** 10.1111/cobi.12050

**Published:** 2013-04-10

**Authors:** Carly N Cook, Michael B Mascia, Mark W Schwartz, Hugh P Possingham, Richard A Fuller

**Affiliations:** * School of Biological Sciences, University of QueenslandBrisbane, Queensland, 4072, Australia; † School of Botany, University of MelbourneParkville, VIC, 3010, Australia; ‡ World Wildlife Fund1250 24th Street NW, Washington, DC, 20037, U.S.A.; § Department of Environmental Science & Policy, 1 Shields Avenue, University of CaliforniaDavis, CA, 95616, U.S.A.

**Keywords:** boundary organizations, boundary science, decision making, environmental management, implementation gap, scientific uncertainty, ciencia de frontera, incertidumbre científica, manejo ambiental, organizaciones de frontera, toma de decisiones, vacío de implementación

## Abstract

There are many barriers to using science to inform conservation policy and practice. Conservation scientists wishing to produce management-relevant science must balance this goal with the imperative of demonstrating novelty and rigor in their science. Decision makers seeking to make evidence-based decisions must balance a desire for knowledge with the need to act despite uncertainty. Generating science that will effectively inform management decisions requires that the production of information (the components of knowledge) be salient (relevant and timely), credible (authoritative, believable, and trusted), and legitimate (developed via a process that considers the values and perspectives of all relevant actors) in the eyes of both researchers and decision makers. We perceive 3 key challenges for those hoping to generate conservation science that achieves all 3 of these information characteristics. First, scientific and management audiences can have contrasting perceptions about the salience of research. Second, the pursuit of scientific credibility can come at the cost of salience and legitimacy in the eyes of decision makers, and, third, different actors can have conflicting views about what constitutes legitimate information. We highlight 4 institutional frameworks that can facilitate science that will inform management: boundary organizations (environmental organizations that span the boundary between science and management), research scientists embedded in resource management agencies, formal links between decision makers and scientists at research-focused institutions, and training programs for conservation professionals. Although these are not the only approaches to generating boundary-spanning science, nor are they mutually exclusive, they provide mechanisms for promoting communication, translation, and mediation across the knowledge–action boundary. We believe that despite the challenges, conservation science should strive to be a boundary science, which both advances scientific understanding and contributes to decision making.

Logrando que la Ciencia de la Conservación Trasponga la Frontera Conocimiento-Acción

**Resumen:**

Hay muchas barreras para utilizar ciencia para informar a la política y práctica de la conservación. Los científicos de la conservación que desean producir ciencia relevante para el manejo deben equilibrar esta meta con el imperativo de demostrar novedad y rigor en su ciencia. Los tomadores de decisiones que buscan que sus decisiones se basen en evidencias deben equilibrar el deseo de conocimientos con la necesidad de actuar a pesar de la incertidumbre. La generación de ciencia que informe efectivamente a las decisiones de manejo requiere que la producción de información (los componentes del conocimiento) sea sobresaliente (relevante y oportuna), creíble (autoritativa, verosímil y confiable) y legítima (desarrollada mediante un proceso que considera los valores y perspectivas de todos los actores relevantes) a la vista tanto de investigadores como de tomadores de decisiones. Percibimos tres retos clave para quienes desean generar ciencia de la conservación que logre estas tres características de la información. Primero, las audiencias científicas y de manejo pueden tener percepciones contrastantes sobre la relevancia de la investigación. Segundo, la credibilidad se puede lograr a costa de la relevancia y legitimidad a la vista de los tomadores de decisiones y tercero, los diferentes actores pueden tener percepciones conflictivas sobre los que constituye información legítima. Resaltamos cuatro marcos institucionales que pueden facilitar que la ciencia informe al manejo: organizaciones de frontera (organizaciones ambientales que trasponen la frontera entre la ciencia y el manejo), investigadores científicos insertados en agencias de manejo de recursos, vínculos formales entre tomadores de decisiones y científicos en instituciones enfocadas a la investigación, y programas de capacitación para profesionales de la conservación. Aunque estos no son los únicos métodos para generar ciencia que traspone fronteras, ni son mutuamente excluyentes, proporcionan mecanismos que promueven la comunicación, traslación y mediación para trasponer la frontera conocimiento-acción. Consideramos que no obstante los retos, la ciencia de la conservación debería pugnar por ser una ciencia de frontera, que incrementa el entendimiento científico y contribuye a la toma de decisiones.

## Introduction

Underpinning conservation policy (regulatory decisions) and practice (on-the-ground decisions) with rigorous scientific evidence can be vital for efficiently solving environmental problems (Pullin & Knight 2001; Sutherland et al. 2004). However, producing science that informs policy and practice is an enduring challenge (Linklater 2003; McNie 2007; Knight et al. 2008). The term *boundary organization* is used to refer to an environmental organization that spans the boundary between science and practice (Guston 2001). Following this definition, we use the term *boundary science* to describe research that both advances scientific understanding and contributes to decision making. This knowledge transfer is bilateral, such that biophysical and social science inform management actions (i.e., evidence-based policy) and management needs inform scientific research (i.e., policy-relevant science). Ideally, conservation science should be a boundary science, and henceforth we refer to conservation science in this ideal form that crosses the boundaries between scholarship and application.

There are obstacles to bridging the knowledge–action boundary. Conservation scientists must balance provision of management-relevant science with the imperative of demonstrating novelty and rigor in their science (Meffe et al. 2006) and are asked to provide science that informs the development of solutions to inherently complex environmental problems (Miller 1993). Impediments to generating boundary-spanning conservation science include a reward structure in science that promotes publication and grant income rather than engaging with conservation practitioners (Gibbons et al. 2008; Arlettaz et al. 2010), journal publication time frames that can be incompatible with solving urgent conservation problems (Meffe 2001), funding constraints preventing questions being addressed at ecologically relevant temporal or spatial scales (Kettenring & Reinhardt Adams 2011), and disincentives within research institutions to conducting the multidisciplinary research necessary to develop realistic solutions to many problems (Ludwig et al. 2001; Knight et al. 2008).

Decision makers responsible for conservation policy and practice must balance a desire for knowledge (information that has been interpreted for their context) with the need to act despite uncertainty (Soulé 1985). Impediments to the use of science cited by decision makers include a lack of financial resources and operational capacity to implement findings (Young & Van Aarde 2011); lack of alignment between the scientific research conducted and the information"/> needed (Fazey et al. 2005; Young & Van Aarde 2011); difficulty accessing and interpreting relevant scientific information (Pullin & Knight 2005; Arlettaz et al. 2010); a perception that scientists are driven by personal agenda and that there is lack of consensus among scientists on the best course of action (Young & Van Aarde 2011); organizational cultures that often do not promote the use of science when implementing management strategies (Young & Van Aarde 2011); and bureaucratic restrictions within agencies. In some cases, it may be more appropriate for decision makers not to incorporate science when innovative approaches to solving environmental problems are impractical to implement, too costly, or their outcomes are not sufficiently predictable (Pannell et al. 2006).

The pervasive challenge of developing science that contributes to both scientific understanding and policy decisions has led to several disciplines coining similar terms. For example, *use-inspired science* is used in medical sciences to describe science that contributes to scientific understanding and clinical practice (Chismar et al. 2011), and *translational science* is used to describe the process of moving scientific discovery to practice. More recently, environmental scientists have adopted the term *actionable science*, which applies to generation of management strategies for environmental problems (Palmer 2012). Spanning the physical and social sciences literatures, Gibbons et al. (1994) describe “mode 2” knowledge production, which is an interactive process used to conduct scientific research in the context of its application. Conservation science that crosses the boundary from scholarship to action can benefit from the ideas generated in other disciplines.

Within the sustainable-development literature, Cash et al. (2003) provide a compelling concept for understanding why some science is translated into action whereas other science is not. They propose that for research to cross the knowledge boundary it must be salient (relevant to decision-making bodies and provided when it is needed), credible (authoritative, believable, and trusted) and legitimate (developed via a process that considers the values and perspectives of all actors) to both scientists and decision makers (Cash et al. 2003). Without all 3 elements, research is likely to be ignored by decision makers. However, not only the nature of the science, but also the perspective from which the science is conducted can affect its relevance for management.

We examined the roles of salience, credibility, and legitimacy in generating science that both advances knowledge and informs policy and practice. Although there is an emerging literature on the need for conservation science to bridge barriers (e.g., Arlettaz et al. 2010), there has been little consideration of the partnerships, institutions, and processes that foster such progress.

## Impediments to Achieving Effective Conservation Science

We perceive at least 3 key challenges for those hoping to achieve boundary-spanning conservation science. First, scientific and management audiences can have contrasting perceptions about the salience of research. Second, the pursuit of scientific credibility can come at the cost of salience and legitimacy of science in the eyes of decision makers, and third, different actors can have conflicting views about what constitutes legitimate information.

### Salience for Scientists Versus Managers

There is a substantial role for science driven solely by the desire for discovery. Curiosity-driven science provides vital building blocks for the application of science and can have unexpected practical relevance (Sutherland et al. 2011). Yet addressing fundamental and novel questions is not always compatible with resolving well-established conservation problems. What is interesting is not always important, and what is important is not always interesting. Boundary scientists seek relevance on both sides of the knowledge–action boundary, a goal that conservation science should strive for (Meffe et al. 2006). To attract funding and facilitate publication in reputable journals, research questions must be novel, but if the research is not relevant to the current problems faced by decision makers it will not influence conservation practice (Linklater 2003). This tension has led to a well-documented mismatch between the types of research appearing in the conservation-science literature and that most relevant to policy and management (e.g., Whitten et al. 2001; Fazey et al. 2005; Knight et al. 2008). Managers report that a lack of research relevant to their needs is a major impediment to the use of science to inform decisions and that irrelevant or unrealistic recommendations can undermine the credibility of scientists (Young & Van Aarde 2011). Salience also involves information being provided in a timely fashion when it is needed for a decision (Cash et al. 2003). However, the urgent and dynamic nature of many conservation problems means that research can be perpetually out of sync with management (Linklater 2003). This issue is exacerbated by the long time frames often required to publish research (Meffe 2001).

### Scientific Credibility Versus Salience and Legitimacy

Scientific credibility is important in management-relevant science, but the pursuit of credibility can compromise the salience and legitimacy of information in the eyes of decision makers. The traditional scientific model seeks credibility through objectivity, hypothesis testing, replication, and repetition (Nowotny et al. 2001). Rigorous scientific methods include the use of experimental controls to establish causation and high levels of replication at multiple spatial and temporal scales, all of which can be difficult to achieve in conservation research (Ferraro & Pattanayak 2006). Methods such as before-after-control-impact (BACI) designs (Bried & Ervin 2011) and credible landscape-level approaches that address conservation problems (e.g., Thompson et al. 2009) can assist in some cases. However, a tendency to simplify research questions to suit rigorous scientific methods can compromise the salience of those questions for decision makers, who must confront the real complexity of environmental problems. Conversely, credible research can lead to highly technical outputs that practitioners find unintelligible (Pullin & Knight 2005), which further impedes the application of research findings even if they are salient.

Achieving credibility by reducing the uncertainty associated with the outcomes of a conservation action has many advantages, such as simplifying decisions and increasing the probability of achieving the stated goal (Sutherland et al. 2004). However, legitimate approaches must account for restrictions to implementation. If achieving high levels of certainty requires long lag times, then the salience of the science for decision makers is reduced. Likewise, management approaches that are too costly to implement lack legitimacy for decision makers. Replication and repetition may incrementally reduce scientific uncertainty, although background environmental variation can make detection of clear environmental trends elusive despite decades of data collection (Magurran et al. 2010). Managers do not always require high levels of confidence to act because delaying action until they are certain they need to act can lead to more expensive management actions (Maguire 1991; Field et al. 2004) or even undesirable outcomes (e.g., the extinction of the Hawaiian Po'ouli [*Melamprosops phaeosoma*] [Black & Groombridge 2010]). Likewise, data collection that diverts funds from on-the-ground management may not always be a good use of resources (Grantham et al. 2009; McDonald-Madden et al. 2010). Therefore, the time and resources required to achieve high levels of certainty can lead to unrealistic recommendations that are not viewed as legitimate by decision makers (Young & Van Aarde 2011), despite their scientific credibility.

The focus on reducing uncertainty can distract from the fact that the acquisition of new knowledge may not materially change what is considered the best course of action. Conservation professionals rarely calculate the value of new information to management, and more research may not always lead to more effective decisions (Runge et al. 2011b). The application of existing knowledge can allow the likely outcomes of management to be predicted with reasonable certainty without expensive data collection. For example, Bayesian methods can quickly reduce uncertainty by combining expert opinion with data (Smith et al. 2007) and have provided timely scientific advice for management decisions (e.g., Punt & Hilborn 1997; Smith et al. 2007). Expert elicitation can also be used to evaluate competing models for how to conduct management. By calculating the expected value of new information, managers can identify when information will be valuable enough to decision makers to warrant additional data collection (Runge et al. 2011b).

**Figure 1:**
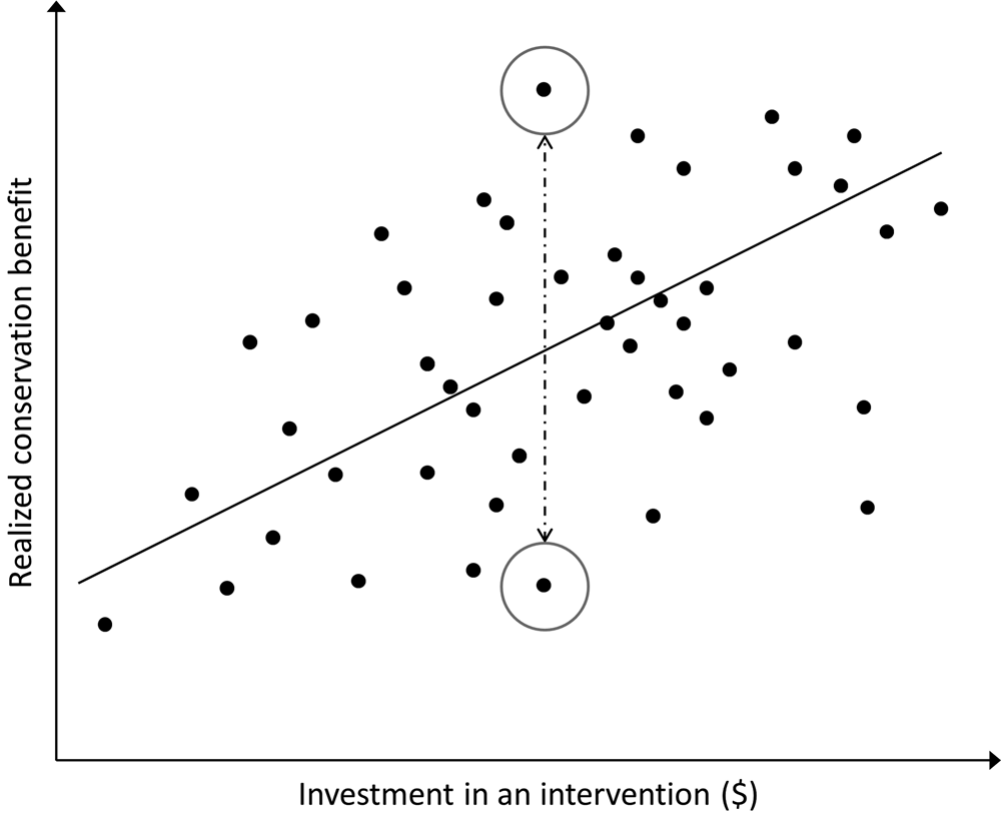
A general relation between monetary investment in a conservation intervention and the desired conservation benefit, which masks substantial heterogeneity in the outcomes at different sites (circled points indicate the degree to which different outcomes can be generated despite the same level of investment in an intervention).

Attempting to develop a single model that predicts management outcomes in all contexts (environmental, social, and political factors relevant to a management decision) can increase scientific credibility (Pullin & Stewart 2006). Unfortunately, such generalizations are rarely adequate (Weiner 1995) because they mask much of the variation in the underlying data that arise from differences among taxonomic groups, geographic locations, and temporal fluctuations (e.g., Bayard & Elphick 2010). When there is heterogeneity in the outcomes of a management intervention, an individual conservation manager may gain little from a general model (Fig. 1). Instead, theory can provide useful heuristics for decisions, such as using existing knowledge about life-history strategies to manage habitat patches for the conservation of birds (Shanahan & Possingham 2009). Although using existing knowledge to develop heuristics requires accepting lower scientific certainty than if specific data were collected on individual species or habitats, and accepting that in some cases the wrong decision may be made, it provides managers with a rational basis on which to act immediately.

An impediment to the production of credible information is that scientific enquiry is a process that fosters debate about the meaning of research outcomes. Although debate is fundamental to science, a lack of consensus among scientists can lead to confusion among those outside the debate and mistrust of researchers among decision makers. These outcomes compromise the perceived credibility of the research findings and the legitimacy of the process of scientific inquiry (Cash et al. 2003; Young & Van Aarde 2011). When action is politically sensitive, such as removing animals from the wild for a captive-breeding program (Clark et al. 1994), decision makers can become paralyzed by uncertainty in research findings and delay necessary action (Ludwig et al. 1993).

### Views of Legitimate Information

Achieving legitimacy for different audiences requires that the values and perspectives of multiple stakeholders and scientific disciplines are represented when developing and implementing research (Cash et al. 2003). However, actors on different sides of the knowledge–action boundary, and across different scientific disciplines, can have different perspectives on what constitutes a legitimate process to produce credible research findings. These views can be deeply held and not easily reconciled (Kleining & Witt 2001). Research findings derived from the use of qualitative research methods, which are often the most rigorous way to study social aspects of conservation (e.g., local ecological knowledge and social effects of conservation interventions), can have low credibility among quantitative researchers but high legitimacy for decision makers. Differing perspectives on legitimate information can impede efforts to include the perspectives and knowledge of some stakeholders in solutions to conservation problems and to develop the multidisciplinary research necessary to provide realistic management approaches (Cash et al. 2003). It is vital to include the perspectives and knowledge of stakeholders, especially decision makers, to ensure that social and ecological research is salient to the management context and legitimate in the eyes of these stakeholders.

## Bridging the Knowledge–Action Boundary

Successful balancing of salience, credibility, and legitimacy benefits from processes such as joint fact-finding (Karl et al. 2007), which exist to engage stakeholders in the process of knowledge production. Approaches to generating boundary science include mechanisms to ensure a collaborative process that represents all stakeholders, facilitates communication across the knowledge–action boundary throughout the research process, translates jargon, and includes mediation between knowledge producers and users (Cash et al. 2003; McNie 2007). Production of effective conservation science can be achieved in a variety of ways, provided there are mechanisms to facilitate communication, translation, and mediation across the boundary.

Institutions and processes that span boundaries are ideally suited to address conservation problems because of the complexity of environmental problems, the need for solutions relevant to multiple stakeholders, contexts, and scientific disciplines, and the diverse users of science that is relevant to both policy and practice. We highlight 4 institutional frameworks that facilitate the science that informs environmental management (Tables 2 & 3): boundary organizations (defined below), research scientists embedded in resource management agencies, formal links between research-focused institutions and resource management agencies, and training programs for conservation professionals. We also discuss how these different approaches to facilitating conservation science can be mixed to harness their different strengths under different circumstances.

### Boundary Organizations

The role of facilitating communication between scientists and decision makers can be assumed by dedicated boundary organizations that operate in both scientific and practical spheres but retain distinct lines of accountability to both groups (Guston 2001). Boundary organizations have been used to address complex environmental problems (Cash et al. 2003) and the interdisciplinary nature of issues such as adapting to climate change (Brooke 2008). There are many boundary organizations that work at the nexus of science, policy, and practice and facilitate communication among them (Table 1), for example, the Ecosystem-Based Management Tools Network (http://www.ebmtools.org), which provides a wide range of training and outreach activities to connect practitioners with tools that incorporate natural and social science into decision making. Nongovernmental organizations that facilitate working groups of scientists, decision makers, and other stakeholder groups to develop management strategies that can be applied across landscapes could also be considered boundary organizations.

**Table 1:** Examples of institutional frameworks that facilitate science that crosses the knowledge to action boundary

Boundary organizations	Scientists embedded in resource management agencies	Formal links between research and practice	Training conservation professionals
Fiji Locally Managed MarineArea Network facilitates a partnership between government authorities, nongovernmental organizations, community leaders, research-focused institutions, and private-sector organizations to protect marine resources	World Wildlife FundConservation Science Program(International)conducts multidisciplinary research to inform on-the-ground programs and communicates findings to other conservation organizations, government agencies and academics	Sulu-Sulawesi Seascape(Philippines) is a collaborationbetween nongovernmentalresource management agenciesand local research-focusedinstitutions to deliverconservation science to informlocal government planning	University of Exeter Master ofScience–Conservation andBiodiversity program is designedwith external resourcemanagement agencies toprovide research and practicalskills, with opportunities forfurther training with resourcemanagement agencies
Healthy Reefs for HealthyPeople (Central America) facilitates partnerships between research-focused institutions, government, and nongovernmental agencies, and the community to improve reef health in the Caribbean	U.S. Department of AgricultureForest Service Research Stations conduct long-term, often spatially extensive research to improve understanding of ecosystems and to provide tools to transfer knowledge into management recommendations	Australian Research CouncilLinkage Grants provides"/> competitive research funding for projects developed as collaborations between resource management agencies and research-focused institutions	Environmental LeadershipProgram Fellowships(International) provide trainingopportunities for midcareerconservation professionals fromall sectors to increase theircapacity and develop networksand leadership skills
Center for InternationalForestry Research(International) conducts and communicates multidisciplinary research to manage forest environments and alleviate poverty	New South Wales Office ofEnvironment and Heritage(Australia) Science Division conducts research at local and landscape levels and provides advice to on-the-ground managers	Grants from philanthropictrusts for conservationresearch, such as the Davidand Lucile Packard Foundation,Gordon and Betty MooreFoundation, WaltonFoundation	Duke Environmental LeadershipMaster of EnvironmentalManagement program providesmidcareer conservationprofessionals withinterdisciplinary scientifictraining in strategicenvironmental-management,communication, and leadershipskills
Resources for the Future(International) conducts and communicates the results of independent, multidisciplinary research to inform environmental policy	Royal Society for the Protectionof Birds (United Kingdom andinternational) conducts research and monitors threatened birds within reserves in the United Kingdom and internationally, which informs policy and management	World Wildlife Fund's FullerScience for Nature Fund provides funding for conservation research and hosts an annual science symposium for decision makers	Leopold Leadership Program advances environmental decision making by providing tenure-track scientists with leadership and communications skills

Establishing separate organizations devoted to promoting the development and use of conservation science is an advantage because these organizations can operate on both sides of the boundary while maintaining their credibility and independence. This independence can bring together groups that may have had poor relationships in the past and can enable boundary organizations to attract funding from a wide range of sources (Guston 2001). However, boundary organizations tend to work best when focused on specific issues in specific places (Osmond et al. 2010). The number of conservation problems and the cost of administering boundary organizations mean that specialized or local organizations will not always be feasible, especially in developing countries.

### Research Scientists in Resource Management Agencies

There are multiple benefits to creating permanent positions that embed research scientists within organizations dominated by decision makers (Jenkins et al. 2012) (Tables 2 & 3). Resource management agencies (government and nongovernmental) can ensure that high-priority knowledge gaps are filled by these researchers, who could provide data about effective interventions and advice relevant to the management context (Young & Van Aarde 2011). Due to their exposure to the management of conservation problems, embedded scientists also have the potential to identify and study conservation problems that have not received scientific attention (e.g., protected area downgrading, downsizing and degazettment [Mascia & Pailler 2011]). Furthermore, scientists can provide in-house expertise for the design and implementation of research and monitoring programs and analysis of data collected by agency staff to ensure decision makers can make informed decisions about compromises between certainty and urgency. Allowing managers to work directly with scientists offers greater potential to apply adaptive-management approaches (e.g., Glen Canyon Dam Adaptive Management Program [Susskind et al. 2012]) that gather salient, credible, and legitimate information from management activities and use that information to guide future decisions.

**Table 2:** The potential benefits and weaknesses of the different approaches to facilitating conservation science for decision makers

Models for facilitating conservation science	Benefits to decision makers	Weaknesses for decision makers
Traditional academic model	rigorous scientific information generated can identify emerging issues and provide unexpected benefits	research may not be relevant or timely
Boundary organizations	increases management-relevant science, provides greater access to existing management-relevant research, and promotes bilateral, active knowledge transfer	requires additional resources, and is not feasible for all conservation problems
Scientists embedded in conservation agencies	increases management-relevant science, provides greater access to existing management-relevant research, provides opportunities to learn from management action (e.g., adaptive management), provides access to tools to aid decisions (e.g., decision theory), and"/> promotes bilateral, active knowledge transfer provides access to expert advice	requires additional resources, and"/> may compromise the quality of research if researchers become isolated from the broader scientific community
Formal links between researchers and decision makers	increases management-relevant science, provides greater access to existing management-relevant research, promotes bilateral, active knowledge transfer, and provides access to expert advice	requires additional resources, and success depends on the commitment of both scientists and decision makers
Training conservation professionals	improves scientific knowledge and skills, provides more scientists with an understanding of management contexts, and promotes bilateral, active knowledge transfer	requires additional resources to train existing staff, and benefits may take time to become widespread

**Table 3:** The potential benefits and weaknesses of the different approaches to facilitating conservation science for scientists

Models for facilitating conservation science	Benefits to scientists	Weaknesses for scientists
Traditional academic model	rigorous scientific information generated, and fits within existing training and current reward structures	research finding may not be implemented
Boundary organizations	promotes bilateral, active knowledge transfer, identifies important research questions, and provides access to additional source of funding	requires additional resources, and is not feasible for all conservation problems
Scientists embedded in conservation agencies	identifies important research questions, increases likelihood that research findings are implemented, and promotes bilateral, active knowledge transfer	can lead to scientists becoming isolated from the academic community, may limit access to the primary literature and research students, and may compromise objectivity and independence
Formal links between researchers and decision makers	identifies important research questions, increases likelihood that research findings are implemented, provides access to additional source of funding, and promotes"/> bilateral, active knowledge transfer	requires time be spent on bureaucratic processes, and success depends on the commitment of both scientists and decision makers
Training conservation professionals	provides a better understanding of management context, and promotes"/> bilateral, active knowledge transfer	requires some content from the traditional syllabus be sacrificed, and curriculum development may divert time from research activities

An additional benefit of embedding researchers in resource management agencies is that a close working relationship between on-the-ground managers and scientists can help overcome the resistance managers sometimes have to using scientific information (Young & Van Aarde 2011). When managers can advise scientists about research priorities and the real-world constraints on management, research is more likely to result in salient and legitimate solutions. Moreover, scientists within resource management agencies could filter, synthesize, and translate the peer-reviewed literature into management approaches. This would overcome the impediments of access and interpretation of literature that can prevent the use of science in practice (Fazey et al. 2005; Pullin & Knight 2005; Arlettaz et al. 2010) and mimic the preference of managers to seek advice from scientists they consider credible (Seavy & Howell 2010).

There are several challenges associated with embedding researchers in resource management agencies. These include the potential for scientists to become isolated from and have the credibility of their research questioned by the wider scientific community. Likewise, achieving salience and legitimacy for policy and practice may lead to compromises in scientific rigor that challenge traditional notions of scientific credibility. For example, decision makers may be willing to accept lower levels of confidence to reduce costs and facilitate timely information for urgent action, or they may favor avoiding type II error (i.e., accepting a false null hypothesis [failing to recognize a genuine problem]) rather than the traditional emphasis on reducing type I error (i.e., rejecting a true null hypothesis [false alarms]) (Shrader-Frechette & McCoy 1992; Field et al. 2004). In these cases it may be necessary for embedded scientists to assume the role of mediators across the boundary and communicate the needs of decision makers to other scientists and provide the information necessary for decision makers to seek compromise between scientific credibility and realistic solutions. To ensure that researchers genuinely operate in both spheres, it is important that they engage with the wider scientific community, for example through professional bodies such as the Society for Conservation Biology (Schwartz et al. 2008) and by participating in peer-review and editorial processes. Other measures to strengthen the benefits from embedding scientists in resource management agencies include full access to the primary literature and participation in the training of conservation professionals (e.g., graduate students who conduct research through formal links with academic institutions).

Embedding scientists in resource management agencies has been a valuable practice for decades, and we are aware of a multitude of conservation organizations in which high-quality research is conducted and scientists are respected globally (Table 1). However, many agencies are downsizing their science divisions and outsourcing research. Reversing this trend would require clear articulation and illustration of the value of science in decision making. In countries where internal conservation budgets fall well short of what is needed for management and science, additional support may be necessary in the form of funding or exchange programs aimed at building scientific capacity.

### Links between Researchers and Decision Makers

Where it is currently impossible to embed scientists in resource management agencies (Jenkins et al. 2012), agencies can still benefit from closer links with scientists. Such links have been developed through formal arrangements between resource management agencies and scientists at research-focused institutions, whereby agencies supply priority research questions and a small financial incentive, such as a research stipend or contribution toward project costs. Ideally, these arrangements are actively managed by individuals who assume the responsibility for communication, translation, and mediation across the boundary. This approach benefits the agency because it provides expertise of scientists from a wide range of disciplines and the enthusiasm and energy of staff or students. The research-focused institutions benefit from the additional source of research funds, and their staff or students are provided with an opportunity to conduct management-relevant research.

Many successful models exist for developing formal links between decision makers and conservation scientists (Table 1). The Research Partners Program operated by an Australian management agency (Parks Victoria) maintains formal agreements with several research-focused institutions whose scientists or graduate students conduct management-relevant research in exchange for a financial contribution to this research. Likewise, the U.S. Department of the Interior has cooperative research units located at land-grant universities. These units link research funds and stipends to management-relevant projects. Alternatively, employees of resource management agencies can work within research-focused institutions to develop and facilitate conservation science. For example, many nongovernmental organizations, such as Wildlife Conservation Society and The Nature Conservancy, have funds for their staff to spend time within academic institutions. Although there are many benefits to these models, the success of these systems relies on the good will of researchers to actively communicate with managers throughout the project and to share research findings.

### Training Conservation Professionals

The growth in formal training courses in conservation (Noss 1997) provides an opportunity to train future generations of conservation professionals to facilitate conservation science. The skills required of conservation practitioners differ from those required of conservation scientists, and existing academic training programs generally fail to provide training in both skill sets (Muir & Schwartz 2009). Training individuals who can effectively operate in both spheres of the knowledge–action boundary, regardless of where they are employed, requires that students be provided with skills relevant to both scientists and decision makers so they can communicate, facilitate, and mediate across the knowledge–action boundary. Several organizations, not just academic institutions, offer such programs (e.g., National Conservation Training Center [Runge et al. 2011a]) (Table 1), although it may be some time before these programs have widespread effects.

Identifying skills required by conservation scientists and decision makers can help develop training programs that teach a combination of these skills (Muir & Schwartz 2009). The balance between the knowledge required by both groups of conservation professionals can be achieved by involving both scientists and decision makers in training programs that teach students about tools that can assist decision makers to act under uncertainty (e.g., decision theory, which is used to identify the optimal decision given limited data or high uncertainty [Possingham 2001; Polasky et al. 2011] and adaptive management, the systematic acquisition and application of information to improve management over time [Holling 1978]). Although it is vital that the scientific training of conservation professionals not be compromised by sacrificing good experimental design and analysis, it is important to ensure training programs also deliver skills, such as the ability to communicate science to decision makers, and an understanding of how policy is generated and implemented (Muir & Schwartz 2009).

### Combined Approaches to Achieving Effective Conservation Science

The 4 approaches for spanning the knowledge–action boundary that we highlight can each be effective at facilitating effective conservation science but are by no means the only methods, nor are they mutually exclusive. For example, using formal links such as internship programs to place researchers in resource management agencies for discrete periods of time does not incur a long-term cost. Internships also educate researchers about operational constraints and organizational cultures and can provide decision makers with opportunities to learn new skills. These arrangements can increase the participation of scientists in advisory committees and lead them to adapt their research programs to fill specific knowledge gaps (Jenkins et al. 2012). Likewise, knowledge brokers, who establish and maintain links between researchers and decision makers by translating research findings (Lomas 1997), can perform the role of boundary organizations. To be a knowledge broker, one requires training in both scientific and decision-making skills. These individuals can operate within boundary organizations or within management or research-focused agencies.

Given the diversity of approaches possible for facilitating conservation science, it is important to evaluate the effectiveness of these approaches and to determine the circumstances under which they will be most successful. Salience, credibility, and legitimacy of conservation research are critical for harnessing existing knowledge, developing realistic recommendations, and improving the uptake of research in conservation policy and practice. Achieving boundary science requires that conservation professionals be prepared to engage individuals across the knowledge–action boundary and the boundaries between scientific disciplines and that they challenge traditional models of knowledge production.
